# Supplementary material for: Transcriptional dynamics of colorectal cancer risk associated variation at 11q23.1 correlate with tuft cell abundance and marker expression in silico
Source: Sci Rep. 2022 Aug 10;12:13609. doi: 10.1038/s41598-022-17887-5 (PMC9365857; doi:10.1038/s41598-022-17887-5)
Supplement: Supplementary file 1 — Supplementary Information 1. [file 41598_2022_17887_MOESM1_ESM.docx]

**Supplementary Material**

| **Subject ID** | **Status** | **Location** | **Gender** |
| --- | --- | --- | --- |
| N8 | Healthy | #N/A | Female |
| N10 | Healthy | Right_Colon | Female |
| N11 | Healthy | Right_Colon | Male |
| N13 | Healthy | Right_Colon | Female |
| N15 | Healthy | Right_Colon | Male |
| N16 | Healthy | Right_Colon | Male |
| N17 | Healthy | Transverse_Colon | Male |
| N18 | Healthy | Transverse_Colon | Female |
| N20 | Healthy | #N/A | Female |
| N21 | Healthy | Left_Colon | Female |
| N46 | Healthy | Right_Colon | Male |

**Supplementary Table S1.** Smillie *et al.,* (22) patient metadata

**
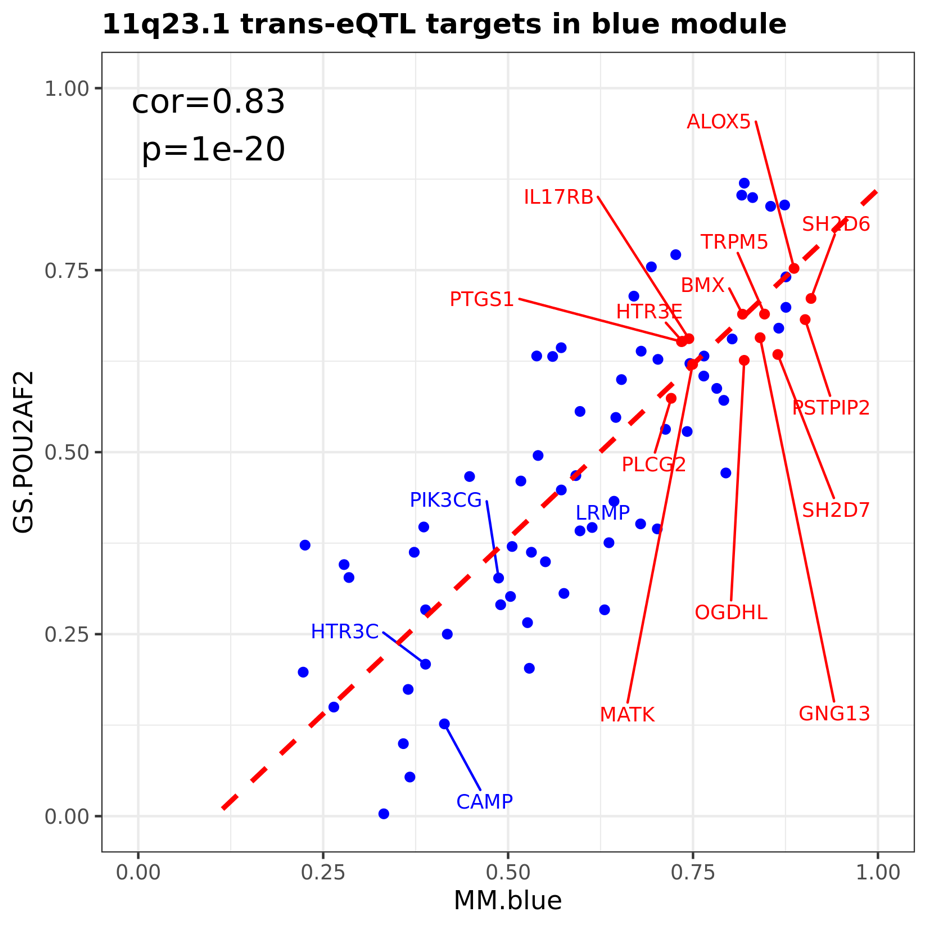
**

**Supplementary Figure S1.** Correlation of Module Membership (MM) and Gene Significance (GS) for *POU2AF2* expression in the blue module. 11q23.1 trans-eQTL targets are highlighted, red if MM.blue and GS.POU2AF2 > 0.5.

| **Gene_symbol** | **GS.POU2AF2** | **p.GS.POU2AF2** | *IKZF4* | 0.528361091 | 0.011477883 |
| --- | --- | --- | --- | --- | --- |
| *MFSD7* | 0.869516061 | 1.49E-07 | *FAM65A* | 0.495417788 | 0.019052761 |
| *P2RY2* | 0.852996421 | 4.57E-07 | *GFI1B* | 0.471411163 | 0.026778703 |
| *FANK1* | 0.849691779 | 5.63E-07 | *SGK223* | 0.467790056 | 0.028134237 |
| *LINC00261* | 0.839485426 | 1.04E-06 | *RNF144A* | 0.466520671 | 0.028622119 |
| *KLK4* | 0.837697653 | 1.15E-06 | *CHSY1* | 0.460356935 | 0.031087422 |
| *PLEKHG4* | 0.771254176 | 2.65E-05 | *DKK3* | 0.448035289 | 0.036517806 |
| *RNF32* | 0.754523148 | 4.97E-05 | *ZFP1* | 0.432558977 | 0.044363985 |
| *ALOX5* | 0.752415106 | 5.36E-05 | *AFAP1L2* | 0.401466056 | 0.064037752 |
| *PTPRD* | 0.740741398 | 8.05E-05 | *TBC1D24* | 0.397206607 | 0.067180992 |
| *ASTE1* | 0.714297945 | 0.000188259 | *LRMP* | 0.396518404 | 0.067699644 |
| *SH2D6* | 0.71110102 | 2.07E-04 | *PODXL* | 0.394606685 | 0.069156315 |
| *HCK* | 0.698902984 | 2.96E-04 | *SLC25A30* | 0.391914382 | 0.071247869 |
| *TRPM5* | 0.689672239 | 3.84E-04 | *ARHGEF3* | 0.375600915 | 0.084958526 |
| *BMX* | 0.689502913 | 3.85E-04 | *ARHGEF2* | 0.372293733 | 0.087962678 |
| *PSTPIP2* | 0.682088126 | 4.71E-04 | *FILIP1L* | 0.370412873 | 0.089706055 |
| *MYOM1* | 0.670290717 | 6.42E-04 | *C20orf194* | 0.362507448 | 0.097315042 |
| *GNG13* | 0.657207652 | 8.90E-04 | *NOTCH4* | 0.362506253 | 0.097316226 |
| *IL17RB* | 0.655879017 | 0.000918972 | *ARHGAP31* | 0.3494408 | 0.11091913 |
| *CYR61* | 0.655513165 | 0.000927169 | *PGAM4* | 0.34556863 | 0.115204208 |
| *HTR3E* | 0.652281 | 0.001002333 | *FGFRL1* | 0.32779629 | 0.136419162 |
| *PTGS1* | 0.651790925 | 0.001014171 | *PIK3CG* | 0.327080559 | 0.137328021 |
| *WWP2* | 0.643465569 | 0.001234253 | *ATF3* | 0.305953301 | 0.16612758 |
| *FYB* | 0.638720231 | 0.001376965 | *PPP1R3B* | 0.301754652 | 0.172314946 |
| *SH2D7* | 0.63416094 | 0.001527043 | *CAPN9* | 0.290378683 | 0.189872811 |
| *SNRPA* | 0.632098175 | 0.00159937 | *APH1B* | 0.283453554 | 0.201136526 |
| *AC025335.1* | 0.632010561 | 0.001602506 | *MT1H* | 0.283442955 | 0.201154103 |
| *RAI1* | 0.631424527 | 0.001623611 | *GNB5* | 0.265793153 | 0.23186717 |
| *MARCKSL1* | 0.627445473 | 0.001773235 | *SNORA31* | 0.249996893 | 0.261829995 |
| *OGDHL* | 0.626140466 | 0.00182478 | *HTR3C* | 0.20872 | 0.351252864 |
| *CRYM* | 0.621805521 | 0.002005201 | *TRIB1* | 0.203124291 | 0.364604113 |
| *MATK* | 0.620641028 | 0.002056158 | *COL1A1* | 0.197820332 | 0.377525123 |
| *ALOX5AP* | 0.604524135 | 0.002881786 | *PIP5KL1* | 0.174089375 | 0.438438491 |
| *RP11-315O6.1* | 0.599692821 | 0.003177792 | *CGNL1* | 0.149829973 | 0.505720737 |
| *MSLN* | 0.587571695 | 0.004034646 | *CAMP* | 0.126754067 | 0.574047366 |
| *PLCG2* | 0.573987877 | 0.005216381 | *ISPD* | 0.099579334 | 0.659282892 |
| *SCRN1* | 0.571174034 | 0.005494069 | *CAPRIN2* | 0.053739047 | 0.812256115 |
| *CD96* | 0.555950868 | 0.007218247 | *DLG5-AS1* | 0.003234775 | 0.988601201 |
| L3MBTL1 | 0.547690051 | 0.008327128 |  |  |  |
| RP11-1220K2.2 | 0.531364238 | 0.010932727 |  |  |  |

**Supplementary Table S2.** Blue module genes, corresponding Gene Significance for *POU2AF2* (GS.POU2AF2) and p-value of Gene Significance for *POU2AF2* (p.GS.POU2AF2). Red=11q23.1 trans-eQTL target (11)

**
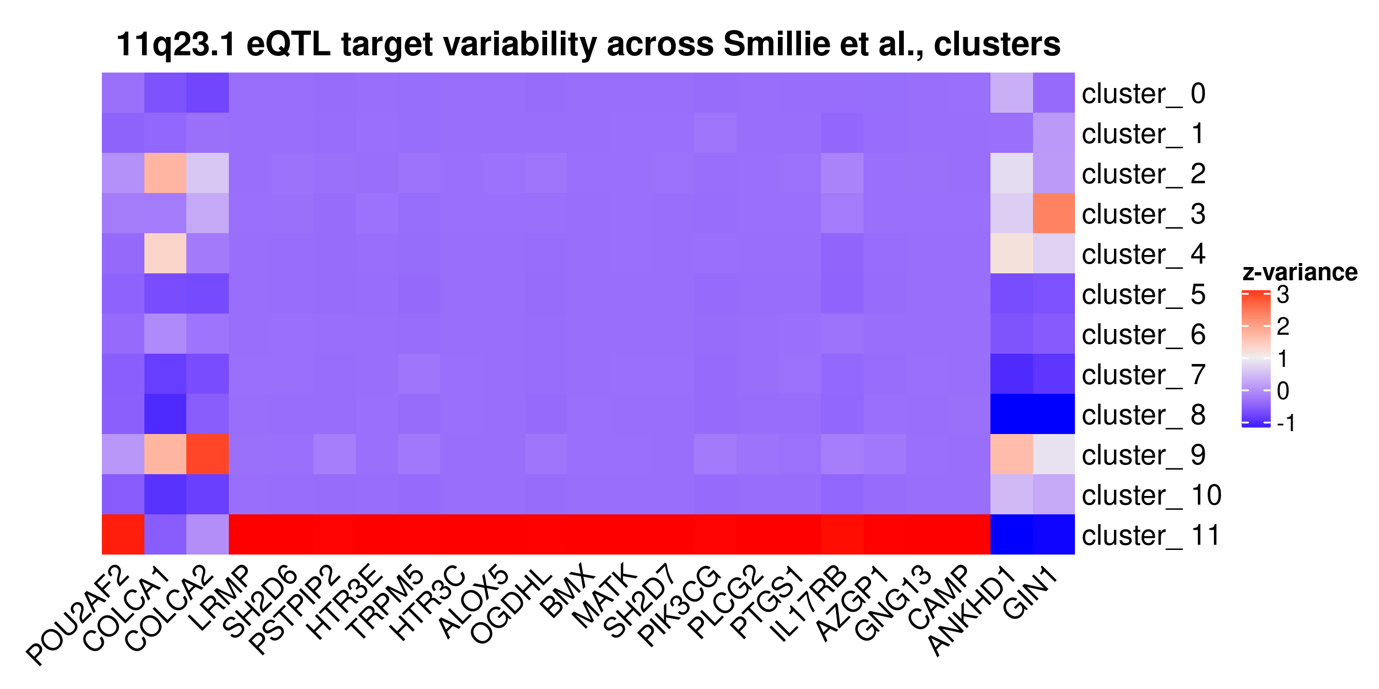
**

**Supplementary Figure S2.** Investigating 11q23.1 eQTL target variance at the single cell level. Variance of 11q23.1 eQTL targets across clusters at the single cell level in the Smillie *et al.,* (22) data. Variance is z-scored within genes.

**
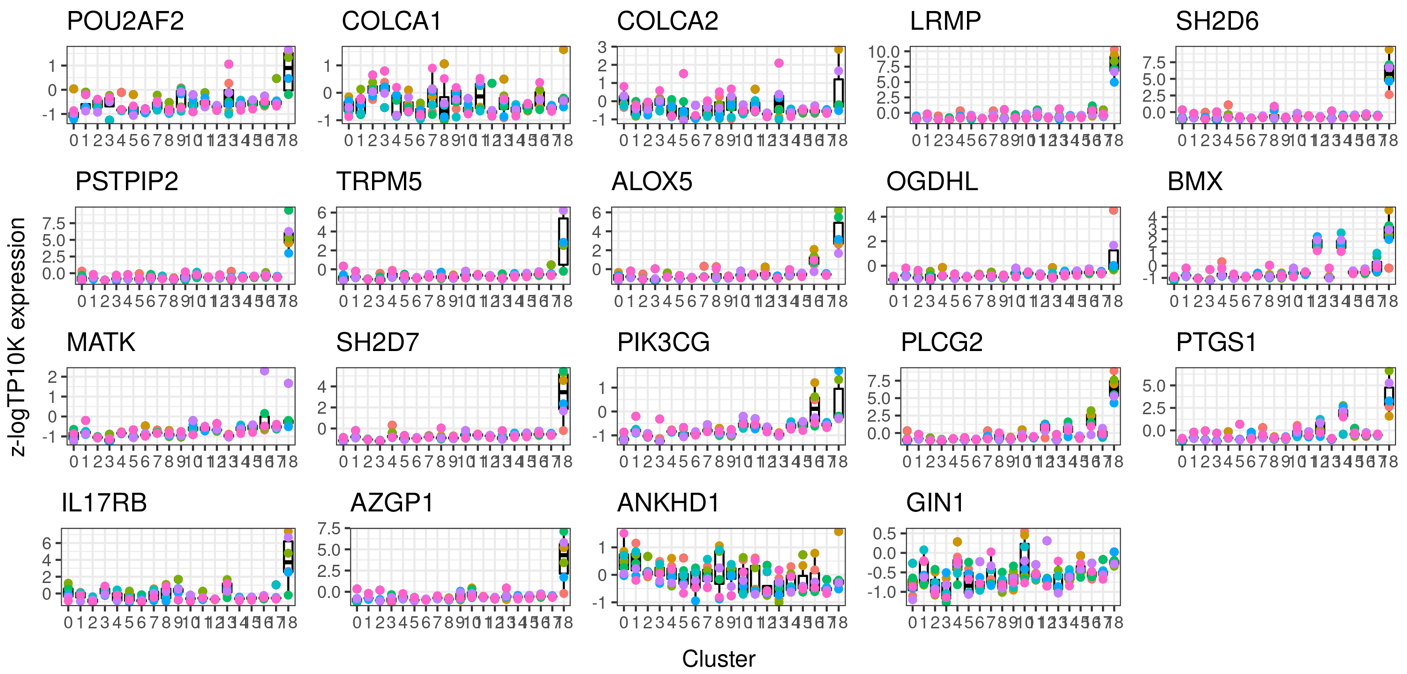
**


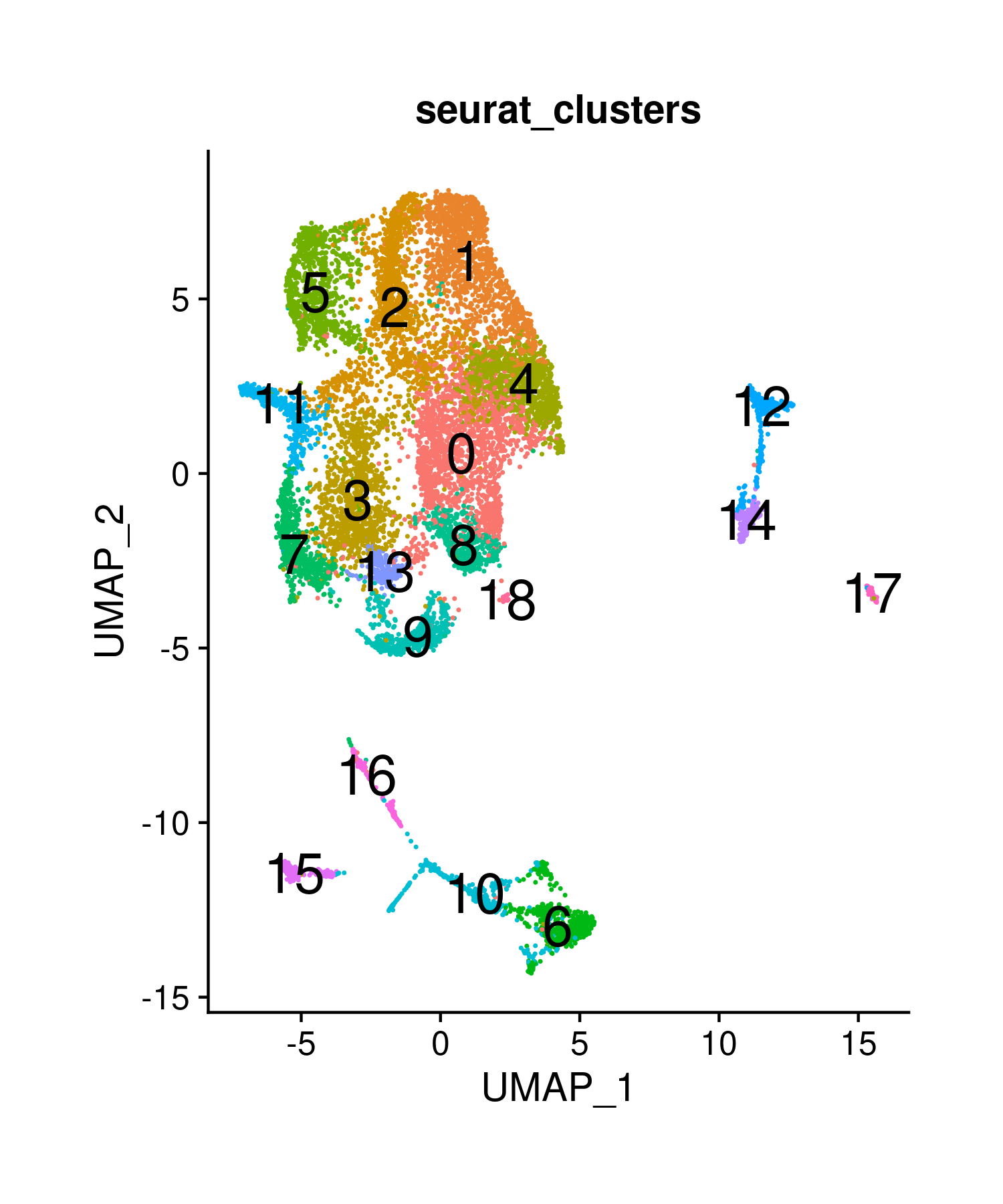


**a**

**b**

**c**

**Trans-eQTL target variability across clusters**


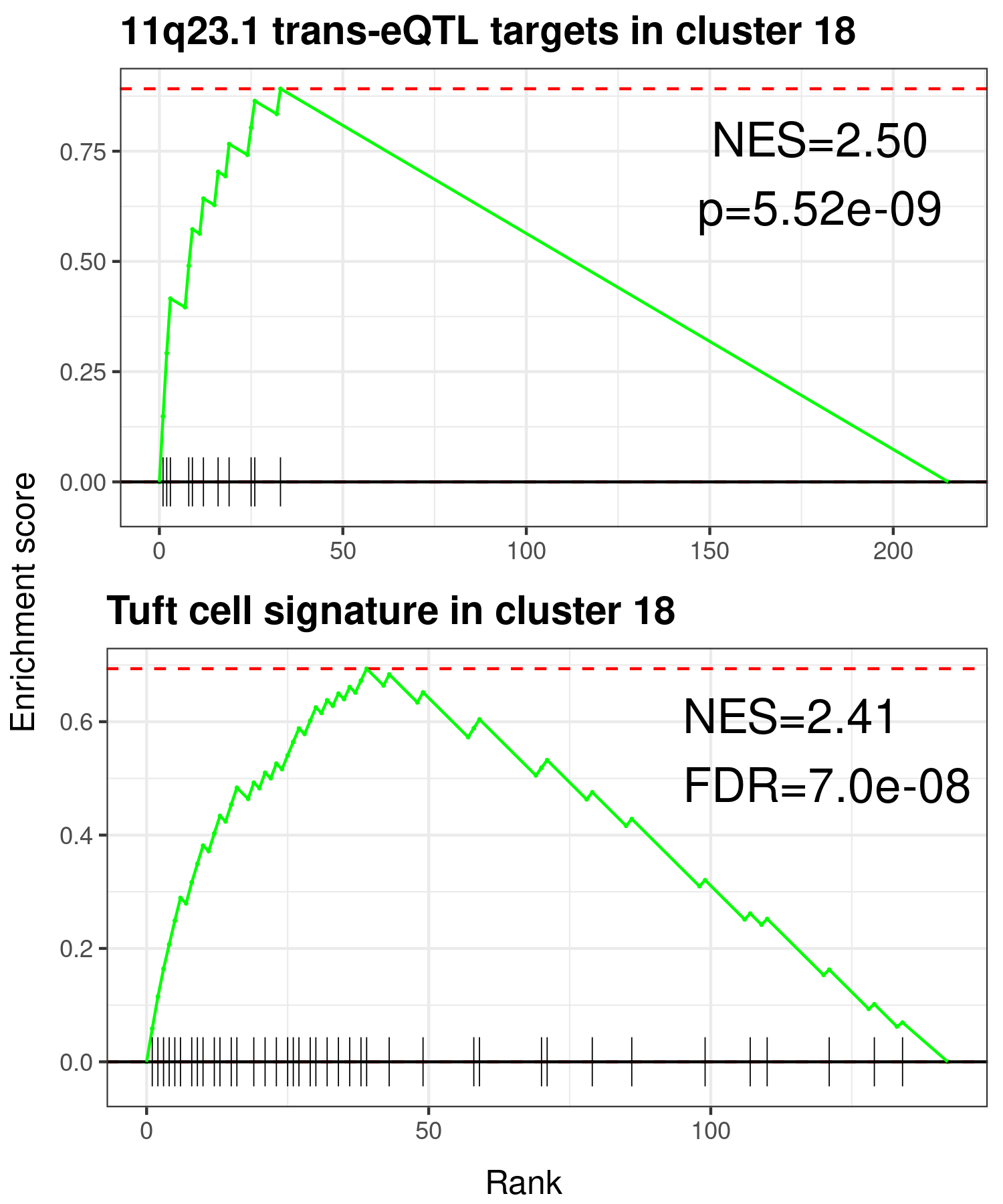


**Supplementary Figure S3. Replication of 11q23.1 eQTL expression mapping in independent dataset.** (A) UMAP of 11,651 healthy colonic epithelial scRNASeq (23). (B) Upper: GSEA of 11q23.1 trans-eQTLs (11; FDR<0.05) in cluster 18. Lower: GSEA of putative colonic tuft cell signature (22) in cluster 18. (D) Relative expression of 11q23.1 trans-eQTLs (FDR<0.05) across clusters**.**

| **Gene** | **p_val** | **avg_log2FC** | **pct.1** | **pct.2** | **p_val_adj** |
| --- | --- | --- | --- | --- | --- |
| *LRMP* | 2.04E-73 | 2.614295875 | 0.903 | 0.004 | 3.19E-69 |
| *IL17RB* | 6.17E-69 | 1.454372429 | 0.516 | 0.033 | 9.64E-65 |
| *SH2D6* | 3.81E-64 | 2.521466548 | 0.806 | 0.002 | 5.95E-60 |
| *PLCG2* | 1.11E-50 | 2.182226523 | 0.71 | 0.011 | 1.73E-46 |
| *PSTPIP2* | 1.31E-45 | 1.656282707 | 0.581 | 0.006 | 2.04E-41 |
| *TRPM5* | 6.51E-44 | 1.403600676 | 0.581 | 0.002 | 1.02E-39 |
| *SH2D7* | 2.90E-39 | 1.090698798 | 0.484 | 0.001 | 4.53E-35 |
| *AZGP1* | 9.92E-39 | 1.061625996 | 0.452 | 0.005 | 1.55E-34 |
| *PTGS1* | 8.37E-30 | 1.282060245 | 0.516 | 0.01 | 1.31E-25 |
| *ALOX5* | 4.14E-29 | 1.32717779 | 0.452 | 0.004 | 6.46E-25 |
| *BMX* | 1.08E-22 | 1.007016149 | 0.419 | 0.012 | 1.68E-18 |

**Supplementary Table S3.** 11q23.1 trans-eQTL targets identified as cluster 18 markers from Elmentaite *et al.,* (23). Markers calculated using MAST (47). Avg_log2FC=average log2 fold change between cluster 11 and all other clusters. Pct1=Proportion of cluster 18 cells expressing gene. Pct2=Proportion of non-cluster 18 cells expressing gene.

**
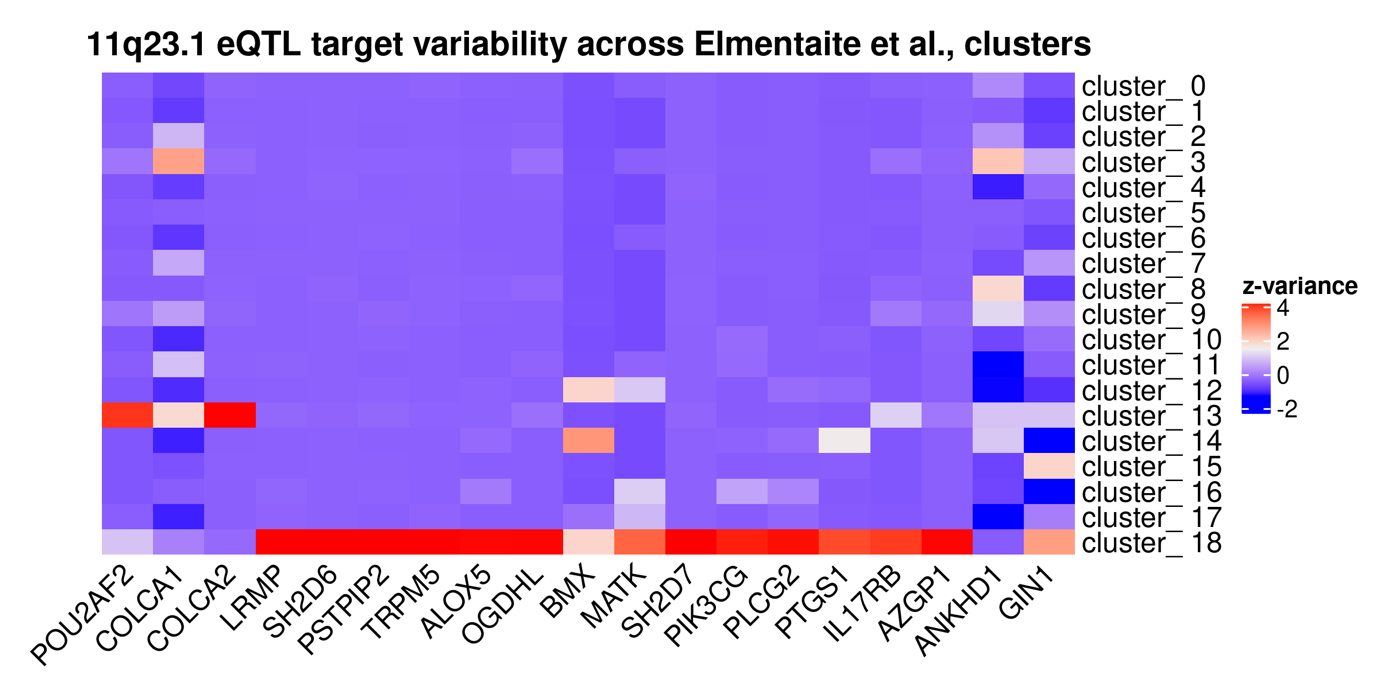
**

**Supplementary Figure S4.** Investigating 11q23.1 eQTL target variance at the single cell level. Variance of 11q23.1 eQTL targets across clusters at the single cell level in Elmentaite *et al.,* (23) data. Variance is z-scored within genes.

| **Sample** | **Subject** | **freebayes_rs3087967** | **samtools_rs3087967** |
| --- | --- | --- | --- |
| Human_colon_16S8123908 | 411C | CC | CC |
| Human_colon_16S8000473 | 417C | T/C | CC |
| Human_colon_16S8000477 | 417C | CC | CC |
| Human_colon_16S8000481 | 417C | CC | CC |
| Human_colon_16S8000479 | 417C | CC | CC |
| Human_colon_16S8000475 | 417C | CC | CC |
| Human_colon_16S8002627 | 432c | CC | CC |
| Human_colon_16S8002630 | 432c | CC | CC |

**Supplementary Table S4.** Elmentaite *et al.,* (23) samples are homozygous non-risk at rs3087967. rs3087967 genotype results of both freebayes (24) and Bcftools (25) variant calling methods. T=colorectal cancer risk allele, C=non-risk allele

| **Gene** | **Smillie_cluster11** | **Elmentaite_cluster18** | **FC** |
| --- | --- | --- | --- |
| *POU2AF2* | 1.275873075 | 0.656341714 | 1.94391587 |
| *COLCA1* | 1.346177989 | NA | NA |
| *COLCA2* | 1.381191066 | 0.834903033 | 1.65431315 |
| *LRMP* | 1.695513426 | 0.173077239 | 9.79628192 |
| *SH2D6* | 0.518050076 | 0.196087378 | 2.64193484 |
| *PSTPIP2* | 0.961252255 | 0.332407278 | 2.89179064 |
| *HTR3C* | 1.850394057 | NA | NA |
| *ALOX5* | 0.855502479 | 0.467755885 | 1.82895075 |
| *OGDHL* | 0.83318323 | 1.141132077 | 0.73013742 |
| *MATK* | 0.815029157 | NA | NA |
| *PIK3CG* | 1.103714386 | 0.73167938 | 1.50846726 |
| *PLCG2* | 0.871942447 | 0.249476603 | 3.49508706 |
| *PTGS1* | 0.673534891 | 0.461933218 | 1.45807849 |
| *IL17RB* | 0.8969865 | 0.467309219 | 1.91947101 |
| *GNG13* | 1.024981369 | NA | NA |
| *CAMP* | 2.260972819 | NA | NA |
| *ANKHD1* | 0.897739169 | NA | NA |
| *GIN1* | 1 | NA | NA |
| *SH2D7* | 0.658046427 | 0.328744382 | 2.00169634 |
| *BMX* | 1.360003183 | 0.360535132 | 3.77217936 |
| *HTR3E* | 0.848772333 | NA | NA |
| *AZGP1* | 1.029538915 | 0.515296922 | 1.99795277 |
| *TRPM5* | 0.599018856 | 0.314108919 | 1.90704186 |

**Supplementary Table S5**. Variability of the majority of 11q23.1 trans-eQTL targets is greater in Smillie *et al.,* (22) cluster 11 compared with Elmentaite *et al.,* (23) cluster 18. Variability analysed within single cells using Seurat *FindVariableGenes* (40). FC = Fold change of variability in Smillie *et al.,* (22) cluster 11 compared with Elmentaite *et al.,* (23) cluster 18

**
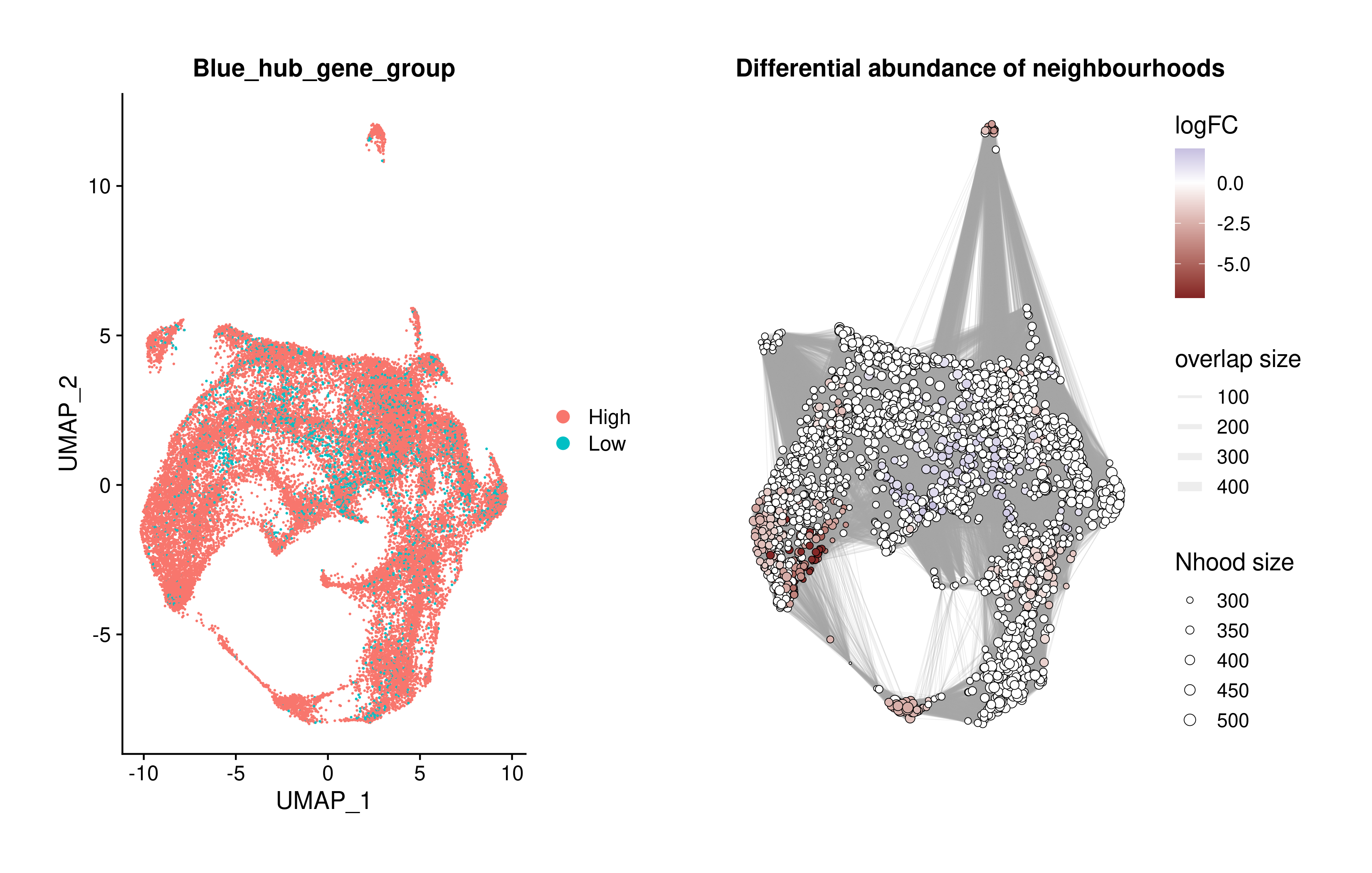
**

**Supplementary Figure S5.** UMAP of cells from each Blue module hub gene group (left) and the differential abundance score of miloR-identified (26) neighbourhoods (right).
